# Oxytocin in Schizophrenia: Pathophysiology and Implications for Future Treatment

**DOI:** 10.3390/ijms22042146

**Published:** 2021-02-21

**Authors:** Kah Kheng Goh, Chun-Hsin Chen, Hsien-Yuan Lane

**Affiliations:** 1 Department of Psychiatry, Wan-Fang Hospital, Taipei Medical University, Taipei 116, Taiwan; havicson@gmail.com; 2 Psychiatric Research Center, Wan-Fang Hospital, Taipei Medical University, Taipei 116, Taiwan; 3 Department of Psychiatry, School of Medicine, College of Medicine, Taipei Medical University, Taipei 110, Taiwan; 4 Graduate Institute of Biomedical Sciences, China Medical University, Taichung 404, Taiwan; hylane@gmail.com; 5 Department of Psychiatry and Brain Disease Research Center, China Medical University Hospital, Taichung 404, Taiwan; 6 Department of Psychology, College of Medical and Health Sciences, Asia University, Taichung 413, Taiwan

**Keywords:** oxytocin, schizophrenia, social cognition, psychopathology

## Abstract

Schizophrenia is a form of mental disorder that is behaviorally characterized by abnormal behavior, such as social function deficits or other behaviors that are disconnected from reality. Dysregulation of oxytocin may play a role in regulating the expression of schizophrenia. Given oxytocin’s role in social cognition and behavior, a variety of studies have examined the potential clinical benefits of oxytocin in improving the psychopathology of patients with schizophrenia. In this review, we highlight the evidence for the role of endogenous oxytocin in schizophrenia, from animal models to human studies. We further discuss the potential of oxytocin as a therapeutic agent for schizophrenia and its implication in future treatment.

## 1. Unmet Clinical Needs in the Treatment of Schizophrenia

Schizophrenia is a syndrome comprised of a collection of signs and symptoms of unknown etiology, predominantly entailing positive symptoms such as paranoid delusions and auditory hallucinations [1]. Apart from positive symptoms, patients with schizophrenia often suffer from negative symptoms accompanied by social dysfunction. These deficits include withdrawal, difficulties with engaging in an interpersonal relationship, lack of involvement in social activities and recreational behavior, and inability to maintain employment [2]. Negative symptoms reflecting reduced motivation for social engagement, including active social avoidance and passive-apathetic social withdrawal, appear to be one of the best predictors for social functioning in patients with schizophrenia [3]. Social dysfunction adversely affects the quality of life and contributes to vulnerability to relapse and worse prognosis in patients with schizophrenia [4].

With the introduction of antipsychotics, improvements in patients with schizophrenia have successfully been achieved by decreasing the admission rate and severity of positive symptoms. Growing evidence indicates that second-generation antipsychotics have not met early hopes for a highly effective treatment for alleviating negative symptoms. Negative symptoms are considered the outward manifestations of social cognitive deficits [5]. Although various pharmacological approaches and psychosocial-oriented interventions have been tailored to improve social cognition and function in patients with schizophrenia, effective treatment for social cognitive deficits remains a clinical obstacle. Reciprocal relationships between social cognition impairment and other psychopathologies of schizophrenia are often discussed. Dysfunctional social cognition leading to false attributions may exacerbate delusions, associated with disengagement from social interaction and blunted motivation for engaging in social relationships, and further genesis of comorbid depression resulting from social withdrawal and anhedonia [6]. Beyond the dopamine hypothesis, several neurotransmitters and neuropeptides, including glutamate, serotonin [7], γ-aminobutyric acid [8], cannabinoid [9], neurotensin, cholecystokinin, corticotropin-releasing factor, neuropeptide Y, and orexin [10], have been implicated in the neuropathophysiology of schizophrenia, predominantly for the underlined negative symptoms and cognitive impairments, in response to the pressing therapeutic need for the development of novel and effective treatment. Among the aforementioned neurotransmitters and neuropeptides, one promising molecule in addressing this need is oxytocin, a hormonal neuropeptide that acts as a neurotransmitter in the brain for regulating social cognition, social affiliation, stress, learning, and memory.

## 2. Oxytocin Fundamentals: Molecules, Physiology, and Function

Oxytocin is a hydrophilic cyclic hormonal neuropeptide consisting of nine amino acids. It was first discovered by Henry Dale in 1906, and its molecular structure was confirmed in 1952. From the oxytocin gene, the inactive precursor oxytocin protein is synthesized and carried by its carrier protein neurophysin I, and then hydrolyzed serially into the active oxytocin nanopeptide, which is released soon after being catalyzed by peptidylglycine alpha-amidating monooxygenase. Oxytocin is mainly metabolized by oxytocinase [11].

Oxytocin is produced by the magnocellular neurosecretory cells of the paraventricular and supraoptic nuclei of the hypothalamus, transported and stored in Herring bodies at the axon terminal, and released by neurohypophysis of the posterior pituitary into the circulation, creating the peripheral effects of parturition and lactation. In addition to peripheral effects, paraventricular nuclei contain magnocellular neurosecretory cells that have specific projections, particularly through axons that collaterally innervate neurons in the amygdala, striatum, substantia nigra, hypothalamus, hippocampus, and nucleus accumbens, where oxytocin receptors are expressed [12]. The oxytocin receptor is known for its diverse functions upon activation with its endogenous ligand oxytocin. In animal studies, oxytocin receptors are localized in the central nucleus of the amygdala and, to a lesser extent, in the medial and basolateral nuclei, as well as in the nucleus accumbens and hippocampus. In humans, expression of oxytocin receptors is noticed in the striatum, substantia nigra, amygdala, and hippocampus [12].

The most intriguing mechanism of the oxytocinergic system is its neuroplasticity and diversity. Taking the modulation of oxytocin in parturition as an example, the oxytocinergic system is self-stimulating and highly open to rapid morphological change [13]. Rapid morphological change in the oxytocin neurons during their physiological function increases the neuroplasticity of the oxytocinergic system. Extensive magnocellular oxytocin neurons are tightly packed in clusters and freed of intervening astrocytic processes in the supraoptic nucleus under the activation process. The juxtaposition allows the oxytocin neurons to be contacted by a larger number of inhibitory and excitatory synapses. Astrocytic processes cover oxytocinergic surfaces and synaptic numbers return to baseline levels once the stimulation is over [14]. In addition to conveying incoming information to the soma, as neurosecretory neurons, dendrites of oxytocin neurons also relay information from the soma back to its own extremities through the release of neuropeptides [15]. The capability of magnocellular neurosecretory neurons of the paraventricular and supraoptic nuclei to regulate their dendritic versus axonal terminal release in either a coordinated or independent manner allows the oxytocinergic system to regulate not only peripherally but also centrally, which differ in significance in human behaviors.

## 3. Implications of Oxytocin in Human Behaviors Associated with Schizophrenia

Social cognition, which refers to mental operations underlying social interaction, is the ability and capacity to understand the thoughts and intentions of others [16], including a range of higher-order (reflective, controlled, and integrative) and lower-order (autonomic, fast) skills [17], subserved by partially dissociable neural networks [18]. Higher-order social cognition skill, for example, theory of mind (also known as mentalizing), refers to the cognitive ability to attribute mental states such as thoughts, beliefs, and intentions that are separate from reality to the self or others, to explain, manipulate, and predict behavior, and to make complex social inferences [17]. The automatic responses of emotional processing, which involves fast cue detection (e.g., eye gaze detection) and judgments (e.g., emotion recognition), are known as lower-order social cognition processes [17]. In other words, social cognition incorporates emotion recognition, attributional style, and theory of mind, which are involved in facilitating social decision-making and associated behaviors [19]. Oxytocin plays a critical role in social cognition. The proposed hypotheses for oxytocin altering human social cognition include direct coupling with dopaminergic neural pathways, mediating reward and reinforcement in the brain, attenuation of defensive behaviors and stress reactivity, and alteration of social information processing by increasing the salience of social stimuli [20]. Increasing interest in this topic has led to the study of the effects of oxytocin on human facial emotion recognition and eye-gaze detection. Intranasal oxytocin administration significantly improves the perception of happy faces [21] by an increased number of fixations and total gaze time [22] but, in contrast, decreases when the face displays an angry expression. In meta-analyses that examined the effects of oxytocin in enhancing emotion recognition, intranasal oxytocin administration enhanced the emotion recognition of faces specifically for the recognition of both happy and fear faces [23,24]. These results support the notion that oxytocin might facilitate social interaction [20] and modulate non-verbal interpersonal communication [21]. Two different oxytocinergic effects have been proposed: the attenuation of early attentional biases toward negative stimuli through downstream effects of amygdala reactivity and hypothalamic–pituitary–adrenal axis stress reactivity, and the enhanced selective attention and recognition of emotional cues by neural circuits that increase social salience and incentive rewards [20]. Notably, the oxytocinergic effects on social cognition in humans are individually different; personal characteristics such as low empathy or social competence may be particularly beneficial [25,26]. Childhood adversities also moderate the effect of oxytocin on social cognition although the findings are inconsistent [20,27].

The role of oxytocin in social bonding is evolving from the initial attention on the mother–infant bonding studies. Oxytocin plays an important role in the development of attachment between infants and parents through early contact and interaction [28]. Besides the early attachment development, a two-week 24 international unit (IU) daily intranasal oxytocin administration to young adult men showed a positive change in attachment from those with avoidant and insecure attachment to a more secure attachment, and increased social responsiveness and quality of life [29]. In terms of social communication, 40 IU intranasal oxytocin administration increased positive communication in relation to negative behavior during a couple of conflict discussions in a randomized controlled trial [30]. The facilitative effect of interpersonal relationships correlates with nonverbal affection, increasing empathy, and trust [31]. Nonverbal affection, facial emotion recognition, and level of in-group trust also improved after administration of a single dose of 24 to 40 IU intranasal oxytocin in human subjects [23,24]. Hurlemann et al. found that 24 IU intranasal oxytocin given 45 min before testing increased emotional empathy but not cognitive empathy in men [32]. The role of oxytocin in emotion, social bonding, and social communication was demonstrated in a resting-state functional magnetic resonance imaging study in which a single dose of 24 IU intranasal oxytocin increased connectivity in the corticostriatal circuitry typically involved those human behaviors [33]. Similarly, intranasal oxytocin increased activity of the amygdala, hippocampus, parahippocampal gyrus, and putamen, and increased connectivity between the amygdala, insula, and caudate during social feedback conditions [34]. However, the involvement of oxytocin in social bonding in humans shows contextual and interindividual variability. Participants performed significantly better under oxytocin treatment when social feedback was given, but showed no response when they received nonsocial feedback [32]. Studies of oxytocin in human behaviors have shed light on the role of oxytocin in pathophysiology and therapeutic potential for patients with schizophrenia whose social cognition is impaired.

## 4. Oxytocin Hypotheses of Schizophrenia in Preclinical Studies

Evidence from animal studies suggests that dysregulation of the oxytocinergic system may play a role in the pathophysiology of schizophrenia. Oxytocin knock-out mice exhibited enhanced deficits in the prepulse inhibition of the startle reflex [35], an endophenotype of schizophrenia that reflects an underlying abnormality in sensorimotor gating of excessive environmental stimuli [36], suggesting that endogenous oxytocin has a protective effect against hypofunction of the glutamatergic system in schizophrenia. The potency of the reversal of prepulse inhibition deficits by antipsychotics is predictive of their potency against positive symptoms. Subcutaneous injection of oxytocin also restores the prepulse inhibition deficits induced by the indirect dopamine agonist (amphetamine) and N-methyl-D-aspartate antagonist (dizocilpine), but not the direct dopamine agonist (apomorphine) [37]. Oxytocin can inhibit excessive mesolimbic dopamine activities, which is supported by subcutaneous injection of oxytocin blocking the hyperactivity in the nucleus accumbens induced by cocaine [38], methamphetamine [39], or phencyclidine [40] in animal studies. These demonstrated a potential therapeutic effect of oxytocin through inhibiting presynaptic dopamine function, subsequently correcting the dopamine hyperactivity in the mesolimbic pathway, which contributes to the positive symptoms of schizophrenia, and counteracting the hypoglutamatergia profile [37].

Modeling negative symptoms in animals is challenging because of the poor understanding of the non-social domains of the negative symptoms of schizophrenia. Nonetheless, efforts have been made to develop appropriate paradigms for various negative symptoms of schizophrenia including social withdrawal [41]. The effects of oxytocin in animal models on the negative symptoms of schizophrenia are mainly focused on social interaction. The study of social interaction in animal models relies on the prairie vole (*Microtus ochrogaster*), which is capable of developing a socially monogamous pair, cooperating to raise their offspring biparentally, and displaying social organization in the mating system [42]. Higher densities of oxytocin receptors in the nucleus accumbens in prairie voles than other non-monogamous vole species [43] and the activation of oxytocin receptors in the nucleus accumbens during the partner preference formation process [44] suggest the involvement of the brain oxytocinergic system in social interaction, particularly in pair-bond formation [45]. RNAi knockdown of oxytocin receptors in the nucleus accumbens inhibits social attachment, alloparental behavior, and partner preference formation in monogamous prairie voles [46], whereas the upregulation of oxytocin receptor expression in the nucleus accumbens after treatment with histone deacetylase inhibitor trichostatin A promotes the formation of partner preference even in the absence of mating [47].

Acute oxytocin administration increases social interaction in animal models. A single infusion of oxytocin into the central nucleus of the amygdala reversed the social interaction deficits induced by phencyclidine in rats [48]. Likewise, marmosets (*Callithrix penicillate*) showed more-often partner-seeking behavior after intranasal oxytocin treatment but reduced proximity to and huddling with their social partner after oxytocin antagonist treatment [49]. The frequency of oxytocin treatment shows different results in social interaction. Most of the acute oxytocin effects on social behavior in animals appear promising [50]. Subchronic or chronic oxytocin administration in animals shows controversial results. Subchronic oxytocin treatment has facilitated the social interaction in mice [51], rats [52,53], and prairie voles [54], but several animal studies showed that the enhancement in social interaction after acute oxytocin treatment diminished if oxytocin was prolonged to chronic administration [55,56]. In most animal studies, oxytocin was administered to normal animals without social deficits; however, the effectiveness of oxytocin in social deficit animal strains has also been proven. For example, chronic oxytocin treatment restored social functioning and increased oxytocin production in the brains of contactin-associated protein-like 2 knock-out mice [57] and Magel2 knock-out mice [58], which both showed a deficit in social behavior. These findings from animal studies have fueled enthusiasm that oxytocin may effectively ameliorate asociality in schizophrenia.

The reciprocal relationship between social cognition deficits and negative symptoms remains under discussion. Social cognition deficits (i.e., difficulty in recognizing emotion and understanding social context) may be associated with disengagement from social interaction and blunted motivation to engage in social relationships [6]. Robust animal studies have examined the implication of oxytocin in social cognition, which is one of the seven specific domains of cognitive impairment in schizophrenia [59]. Both oxytocin knockout mice [60,61,62] and oxytocin receptor knockout mice [63,64] exhibit social recognition deficits; an infusion of oxytocin into the medial preoptic area of mice restores these deficits [61,65]. Oxytocin receptor knockout mice show impaired spatial memory and reduced cognitive flexibility in the T-maze task as the inability to alter their behavior during the reversal phase [66]. The oxytocin treatment significantly modulates social perception by reducing the attention to a negative facial expression [67] and to social reward by promoting prosocial donation behavior [68] in rhesus monkeys (*Macaca mulatta*). Administration of oxytocin also facilitates the prepulse inhibition [56,69], facilitates latent inhibition [70], and improves spatial memory, learning [58,71,72], and novel object recognition [73] in animal studies. From the above preclinical studies, we suggest that oxytocin may be related to the pathophysiology of schizophrenia and may be a potential treatment for patients with schizophrenia.

## 5. Pathophysiology of Schizophrenia: Focus on the Role of Oxytocin

Evidence from animal models suggests that dysregulation of the oxytocinergic system may play a role in the pathophysiology of schizophrenia. A hypothetical neurofunctional model posits that abnormal dopaminergic and oxytocinergic reward system signaling in the amygdala engenders a neural milieu that improperly assigns emotional salience processing and leads to misguided social responses, ranging from withdrawal and isolation to suspicion and paranoia in schizophrenia [12]. Neuroimaging studies examining central oxytocinergic activities revealed that several brain regions may be responsible for the pathophysiology of schizophrenia. Oxytocin increases the connectivity between ventral striatal and pallidal nodes with upstream frontal regions but decreases the strength of downstream pathways between the dorsal striatum and posterior cerebellum for shaping goal-directed behavior [74]. Oxytocin-sensitive basal ganglia networks overlapping those areas sensitive to dopaminergic modulation provide indirect supporting evidence that interactions between these systems might underlie the regulatory function of oxytocin in the domains of salience processing, approach, and motivation [74]. Basal oxytocin levels are generally associated with the activities of the middle and superior frontal cortex, cingulate cortex, cerebellum, and thalamus in schizophrenia [75]. Oxytocin attenuates activities in the amygdala [76], inferior temporal, fusiform and parahippocampal gyri, premotor cortex, middle frontal gyrus, and anterior cingulate gyrus [77], but enhances activities in the middle occipital gyrus, inferior occipital gyrus, and superior temporal gyrus [77], affecting fearful facial emotion recognition in patients with schizophrenia.

The links between the endogenous oxytocinergic system and the deficits in schizophrenia remain limited. Multiple studies have examined the endogenous central and peripheral oxytocin levels in patients with schizophrenia, reporting mixed results. The majority of studies examined the endogenous oxytocinergic system in schizophrenia using the peripheral oxytocin levels as an indicator of central oxytocin function, which may raise questions about the accuracy of oxytocin measurement and its implications. The plasma oxytocin level may also be affected by peripheral organs such as the heart, gastrointestinal tract, and reproductive organs [78]. The validity of using the plasma oxytocin level as a proxy for central oxytocin remains unconfirmed, but a positive association exists between central and peripheral oxytocin levels, although both compartments of the oxytocin system are anatomically separated by the blood–brain barrier [79].

### 5.1. Comparisons to the Healthy Population

Results from the study of endogenous oxytocin levels in schizophrenia are mixed. Endogenous peripheral (measured in serum or plasma) oxytocin levels in patients with schizophrenia are lower than those in the healthy population [80,81,82,83,84,85], although several studies reported opposite findings [86,87] or no differences [88,89]. Two studies published in the 1980s found that cerebrospinal fluid oxytocin levels are higher in schizophrenia than in the healthy population [90,91], but another study found no difference in levels between individuals with or without schizophrenia [92].

### 5.2. Positive Symptoms

The association of endogenous oxytocin levels with positive symptoms of schizophrenia has been proven. Rubin et al. first reported that patients with higher serum oxytocin levels had lower scores on positive symptoms [93]. Similarly, more recent studies replicated the negative correlation between endogenous oxytocin levels and positive symptoms [82,89,94], indicating that lower endogenous oxytocin levels are associated with more severe positive symptoms in patients with schizophrenia. In contrast, Rubin et al. found a significant positive correlation between endogenous oxytocin levels and positive symptoms in probands with schizophrenia, schizoaffective disorder, psychotic bipolar disorder, and their first-degree relatives without a history of psychosis [95]. The difference in the chronicity of the included study populations may account for the opposing results. Another possibility is that oxytocin may have a protective effect when the range of oxytocin values is low, but detrimental effects when oxytocin values are too high [95].

### 5.3. Negative Symptoms

An inverse relationship between endogenous oxytocin levels and the negative symptoms of schizophrenia has been consistently demonstrated in clinical studies. Both cerebrospinal fluid and plasma levels of oxytocin exhibit a significant negative correlation with negative symptoms in patients with schizophrenia [81,86,93,96,97]. Strauss et al. found that lower plasma oxytocin levels are associated with greater severity of asociality in patients with schizophrenia [86], in agreement with the prediction in symptoms of preoccupation, emotional withdrawal, and passive/apathetic social withdrawal [81]. Kéri et al. also found that low oxytocin levels measured after trust-related interactions significantly predicted negative symptoms of schizophrenia, suggesting that decreased trust-related oxytocin release is related to negative symptoms and may be associated with social withdrawal, isolation, and flattened affect in patients with schizophrenia [97].

### 5.4. Cognitive Functions

Goldman et al. first reported that greater accuracy of facial emotion recognition is associated with higher endogenous oxytocin levels in patients with schizophrenia [98]. Higher endogenous oxytocin levels have been proved to result in better facial emotion recognition in schizophrenia in other studies [87,99]. Lower endogenous oxytocin levels are predictive of poor social cognitive functioning [89]. Patients with schizophrenia with higher endogenous oxytocin levels exhibit greater avoidance of negative emotions [100]; however, the reactivity of oxytocin to experimental negative emotions exposure is inversely correlated with cognitive empathy [101,102]. In the metacognition domains, lower endogenous oxytocin levels predict a poorer function in self-integration, perspectives on others, and abilities to respond to psychological problems in patients with schizophrenia [83]. Better social cognitive capacity in patients with schizophrenia with higher endogenous oxytocin levels contributes to a more accurate encoding of socially relevant information [103] and more prosocial behaviors [93]. Apart from social cognitive deficits, endogenous oxytocin is also associated with non-social cognitive deficits. Although failing to find differences between endogenous levels in patients with schizophrenia and a healthy population, lower endogenous oxytocin levels have been associated with poorer processing speed and working memory in patients with schizophrenia [84,104].

### 5.5. Polymorphisms of Oxytocin Gene and Oxytocin Receptor Gene 

The polygenic architecture of schizophrenia comprises both common and rare genetic variants that imply a pathobiological role in the disturbances of neuronal transmission and excitability, neurodevelopment, and the hormonal system [105]. The oxytocin gene and oxytocin receptor gene are important for the regulation of the oxytocinergic system and may contribute to the pathophysiology of schizophrenia. The oxytocin gene is located at chromosome 20, spanning 1 kb over three exons, whereas the oxytocin receptor gene is at chromosome 3, spanning 19 kb with four exons [106]. Single-nucleotide polymorphisms (SNPs) of the oxytocin gene, including rs4813625 [107,108], rs3761248 [107], and rs2740204 [107,108], and the oxytocin receptor gene, including rs53576 [109], rs237885, [109], and rs9840864 [110], may contribute to schizophrenia vulnerability. In addition to disease vulnerability, SNPs of oxytocin receptor genes have been reported to be associated with clinical symptomatology in schizophrenia, including rs237885 [106] and rs237887 [106] with overall symptoms rs237902 [109] with negative symptoms, and rs53576 [109] and rs2254298 [111] with general psychopathology of schizophrenia. Growing evidence indicates the association of oxytocin receptor gene variants with the specific symptoms of schizophrenia, such as oxytocin receptor gene variants rs53576 with emotional withdrawal [112], rs2254298 with empathic concern [111], and rs2268493 with poor performance on social cognition index, mentalizing, and social perception [113]. Not only clinical symptomatology but also treatment response are reported to be associated with genetic variants of the oxytocin receptor gene, such as the effects of antipsychotics on positive symptoms being predicted by the oxytocin receptor gene variants rs11706648, rs4686301, and rs237899 [106]. In a postmortem study, downregulation of oxytocin receptor mRNA in the temporal cortex was found in patients with schizophrenia [114]. These shreds of evidence may provide clues for understanding the role of oxytocin regulation in patients with schizophrenia.

## 6. Exogenous Oxytocin in Schizophrenia: Myth or Miracle?

In addition to the evidence of the role of endogenous oxytocin in the pathophysiology of schizophrenia, exogenous oxytocin administration in patients with schizophrenia shed light on its implication in the treatment of schizophrenia. With bidirectional functional interactions between oxytocin and dopaminergic systems [115], a number of studies have investigated the potential of oxytocin to treat patients with schizophrenia. Bujanow first described the acute antipsychotic effects of oxytocin in patients with schizophrenia in the early 1970s [116,117]. The oxytocin administered by Bujanow was provided in doses of 10 to 20 IU intravenously or 20 to 25 IU intramuscularly once daily for six to ten injections to produce a rapid therapeutic effect and prevent hospitalization [117]. Different from Bujanow, most of the studies conducted administered oxytocin intranasally to patients with schizophrenia, except for Marotta et al., in which oxytocin was sublingually augmented to clozapine-treated patients with schizophrenia [118]. Studies of augmentation with oxytocin in antipsychotics-treated patients with schizophrenia are summarized in Table 1. Most of the studies were conducted in double-blind, randomized controlled trials, except Ota et al., who designed an open-label protocol [119] and Marotta et al., who retrospectively reviewed medical charts [118]. Oral dosing and parenteral injections of oxytocin are not considered viable administration routes as the oral route causes inactivation of oxytocin in the gastrointestinal tract and first-pass metabolism in the liver, whereas the parental injections are unlikely to cross the blood–brain barrier and are associated with severe cardiovascular side effects at high doses [13]. Nevertheless, adjunctive intranasal oxytocin in patients with schizophrenia produced no group differences in discontinuation due to any reason and adverse drug reactions compared with the placebo group [120].

The outcomes of augmentation with oxytocin in antipsychotic-treated patients are classified into positive symptoms, negative symptoms, and other cognitive deficits in the following sections.

### 6.1. Positive Symptoms

Most studies conducted have assessed the effects of oxytocin administration on positive symptoms, reporting mixed results. To date, most oxytocin administration studies in humans have been conducted as randomized controlled trials to primarily investigate the ability of oxytocin augmented to a stable dose of antipsychotics to reduce psychotic symptoms in schizophrenia. Feifel et al. conducted a clinical proof-of-concept study of the therapeutic potential of oxytocin, administering 40 IU of intranasal oxytocin twice daily for three weeks to schizophrenia patients, and reported that oxytocin significantly reduced the Positive and Negative Symptoms Scale (PANSS) positive subscale scores [121]. Concordantly, randomized controlled trials demonstrated a significant improvement in the positive symptoms of patients with schizophrenia after intranasal oxytocin treatment [122,123]. An open-label study that provided a three-month chronic intranasal oxytocin supplement with 12 IU twice daily for patients with schizophrenia demonstrated a significant reduction in the PANSS positive subscale scores [119]. A different delivery method, augmented with sublingual oxytocin 10 IU once daily to 20 IU thrice daily for at least one year, helped patients with schizophrenia maintain lowered positive symptoms, based on a retrospective chart review [118]. Studies with positive findings have suggested the ability of oxytocin to treat the positive symptoms of schizophrenia. Overall, studies that demonstrated a beneficial effect on the positive symptoms in patients with schizophrenia administered 24 to 40 IU oxytocin intranasally twice daily for two to eight weeks.

Nevertheless, several negative findings of randomized control trials have also been reported. Two studies reported that intranasal oxytocin treatment, 24 IU twice daily for 6 [124] and 12 weeks [19], showed improvement in PANSS positive subscale scores in patients with schizophrenia in both intervention and placebo groups but between-group changes were nonsignificant. Several randomized control trials failed to reveal a positive effect of intranasal oxytocin supplement on the positive symptoms of schizophrenia [125,126,127,128,129,130]. These studies were conducted for 6 to 16 weeks and provided intranasal oxytocin at doses of 20 to 40 IU once or twice daily to patients with schizophrenia. Notably, most of the aforementioned studies with negative findings were not primarily designed to investigate the effect of oxytocin on the positive symptoms of schizophrenia, and most of them measured the improvement in the symptoms using the Brief Psychiatric Rating Scale (BPRS).

### 6.2. Negative Symptoms

The implications of oxytocin in the negative symptoms of schizophrenia have more often been the focus of clinical studies. In a randomized controlled trial, Feifel et al. first reported that twice-daily 40 IU intranasal oxytocin administration for three weeks [121] significantly decreased the PANSS negative subscale scores in patients with schizophrenia. The same positive findings of the efficacy of oxytocin on the negative symptoms of patients with schizophrenia were observed in following studies that provided intranasal oxytocin at doses of 24 to 40 IU twice daily for 6 to 12 weeks [19,122,124,131]. Lee et al. found that the inpatient but not the outpatient subgroup of patients with schizophrenia benefited from 20 IU intranasal oxytocin given twice daily for three weeks in the outcomes measured by the Scale for the Assessment of Negative Symptoms (SANS) scores [130]. In an open-label study, Ota et al. also reported a significant improvement in blunted affect, emotional withdrawal, and lack of spontaneity and flow of conversation after 12 IU oxytocin was given intranasally twice daily for three months to patients with schizophrenia [119]. In a retrospective chart review study, Marotta et al. proved the efficacy of augmentation with 10 IU once daily to 20 IU thrice daily for at least one year on the reduction in negative symptoms in patients with schizophrenia [118]. A single dose of 40 IU intranasal oxytocin increased the resting-state functional connectivity between the amygdala and left middle temporal gyrus, superior temporal gyrus, and angular gyrus, which contributes to the negative symptoms of schizophrenia [132].

Several randomized controlled studies failed to detect a significant improvement in negative symptoms after oxytocin administration compared with the placebo group [123,125,126,127,128,129,133]. Although augmentation of oxytocin did not add to the improvement in SANS scores, Cacciotti-Saija et al. revealed that the increased use of oxytocin nasal spray in patients with schizophrenia was positively correlated with the reduction in negative symptoms [126]. Most studies used SANS to assess negative symptoms and provided intranasal oxytocin at 24 to 48 IU twice daily for 2 to 16 weeks.

### 6.3. Cognitive Functions

A growing body of evidence indicates that exogenous oxytocin administration may improve social cognition and neurocognition in patients with schizophrenia. The measurements of social cognition and neurocognition domains are heterogeneous. Compared with the studies that primarily examined the positive and negative symptoms of schizophrenia, most of the studies of social cognition and neurocognition provided intranasal oxytocin in a single dose rather than chronic administration. Various social cognition skills, including the theory of mind in the higher-order and facial emotional recognition in the lower-order social cognition process, have been applied to measure the improvement in social cognition in patients with schizophrenia after oxytocin treatment.

## 7. Lower-Order Social Cognition Process

In the measure of eye gazing in patients with schizophrenia, intranasal oxytocin increased fixation time [134], numbers of fixations, dispersion, and saccade amplitudes [135]. In the domain of facial emotion recognition, Averbeck et al. found that a single dose of 24 IU intranasal oxytocin improved the hexagon emotion discrimination task in patients with schizophrenia [136]. Similar results have been reported in the study of fear recognition [124,137,138], although Fischer-Shofty et al. failed to demonstrate between-group differences in patients with schizophrenia and healthy controls [138]. The effects of oxytocin on the neural response to facial expressions in patients with schizophrenia has also been studied. Amygdala activity decreased for fearful emotion and increased for happy faces in patients with schizophrenia after a 40 IU intranasal oxytocin administration [76]. In most of the studies with positive findings, intranasal oxytocin was given once at 10 to 40 IU, except for Gibson et al., who provided 24 IU intranasal oxytocin twice daily for six weeks [124]. Despite the promising findings, several studies failed to yield a significant improvement in facial emotional recognition after oxytocin treatment [126,129,139].

## 8. Higher-Order Social Cognition Process

The evaluation of the higher-order social cognition process focuses on the cognitive ability to attribute thoughts, beliefs, and intentions that are separate from reality to the self or others to make complex social inferences. Different assessment tools have been used in the randomized controlled trials of oxytocin treatment in patients with schizophrenia. A 40 IU intranasal oxytocin significantly improved the higher-order social cognition composite, including sarcasm, deception, and empathy, in patients with schizophrenia compared with a placebo [140]. Several studies showed improvement in higher-order social cognition, particularly in the faux pas recognition task, which reflects the theory of mind, in patients with schizophrenia, after a single dose of 40 IU intranasal oxytocin [17,141,142], except Pedersen et al., who provided 24 IU intranasal oxytocin twice daily for two weeks [123]. The neural effect of intranasal oxytocin on the theory of mind was proved by increased activation of the right temporo-parietal junction in patients with schizophrenia [142]. Both require inferring the emotions of others in psychological processing; the affective theory of mind is similar to the cognitive component of empathy [143]. In the evaluation of empathy in schizophrenia, both treatment with a single 24 IU dose [144] and chronic 40 IU twice weekly for six weeks [128] of intranasal oxytocin showed an improvement in empathic accuracy in patients with schizophrenia. In social perception, a single intranasal dose of 24 IU oxytocin improved the recognition of kinship in patients with schizophrenia [145]. In a more specific task in social perception, chronic 24 IU intranasal oxytocin administration for 6 [124] and 12 weeks [146] improved the perspective-taking in patients with schizophrenia. Recent studies aimed to examine the improvement in social cognition by measuring the neural processing in the mirror neuron system (MNS) in patients with schizophrenia. Singh et al. found that 48 IU but not 24 IU intranasal oxytocin administration enhanced mu-suppression, i.e., the neural processing in the MNS that is associated with improved social cognition, in male patients with schizophrenia [147]. Similar positive effects of intranasal oxytocin at doses of 36 and 48 IU on mu-suppression were also revealed by Wynn et al. [148]. Notably, most of the studies with positive findings in higher-order social cognition treated the patient with schizophrenia with single dose of 40 IU intranasal oxytocin. Not all studies detected positive results of oxytocin compared with the placebo in the theory of mind concept [19,126,149], social cognition [139,150], social motivation [151], social affiliation [152], and empathy [146] in schizophrenia patients.

## 9. Neurocognition

Few studies focused on the neurocognition effects of oxytocin in patients with schizophrenia. Feifel et al. found that a three-week, twice-daily, 40 IU intranasal oxytocin treatment improved the verbal memory measured by the California Verbal Learning Test but not the working memory measured by the letter number sequence in patients with schizophrenia [153]. However, Michalopoulou et al. found that the working memory measured by Digit Span Backwards Task improved after 24 IU intranasal oxytocin administration [154]. Improvement in verbal fluency measured by Brief Assessment of Cognition in Schizophrenia was found in an open-label study that provided 12 IU oxytocin intranasally twice daily for three months in patients with schizophrenia [119]. Other studies that examined the therapeutic role of oxytocin in the neurocognition of patients with schizophrenia failed to yield a significant result, including studies that measured the letter number sequence [155], Measurement and Treatment Research to Improve Cognition in Schizophrenia (MATRICS) Consensus Cognitive Battery composite score [125,128], and Repeatable Battery for the Assessment of Neuropsychological Status [17].

## 10. Miscellaneous

Bradley et al. found that after intranasal administration of 40 IU oxytocin, both schizophrenia patients and healthy controls showed increases in bidding motivated by preferences for both monetary and social reward, but the social reward behavior was significantly different in patients with schizophrenia compared to healthy controls. Overbidding was less initially but sustained across the trials compared with healthy controls [156]. Deficits in olfactory identification have been widely reported in patients with schizophrenia and are associated with negative symptoms [130]. Olfactory identification in patients with schizophrenia has been improved after intranasal oxytocin treatment at a single dose of 40 IU [157] and 20 IU twice daily for three weeks [130].

## 11. Implications for Future Treatment

Recent investigations highlighting the role of oxytocin in the pathophysiology of schizophrenia, along with its tolerability when administered intranasally, have led to enthusiasm about its therapeutic potential. Despite these encouraging preliminary findings, the potential of oxytocin to alleviate symptoms of schizophrenia remains an open question. Augmented with antipsychotics, chronic administration of intranasal oxytocin showed significant improvement in the positive and negative symptoms of schizophrenia, whereas the majority of the improvements in cognitive function resulted from a single dose of intranasal oxytocin. The dosages, frequency, and duration of oxytocin administration differ among studies. The dosages of oxytocin used in these clinical studies ranged from 10 to 84 IU, the frequency from a single dose to thrice daily, duration from once to a year. Besides the differences in dosing and administration protocols, the use of various clinical assessment tools and individual factors affecting oxytocin sensitivity have likely contributed to the heterogeneity both between and within studies. For instance, studies that measured the positive and negative symptoms using PANSS scores were more likely to show promising results than those using BPRS scores and SANS scores in assessment for efficacy of intranasal oxytocin augmentation. The differences and complexity in the measurements of cognitive functions in patients with schizophrenia hinder the comparison of these studies, preventing drawing conclusions. The optimal strategies for oxytocin administration, including dosage, frequency, and duration, and the features of psychopathology or characteristics of patients that might be associated with beneficial effects of oxytocin need to be further studied in the future.

The significant heterogeneities present in the clinical studies pertaining to efficacy outcomes are not surprising given the pathophysiology underpinnings of both schizophrenia and human oxytocinergic system being inadequately characterized at this point [158]. With bidirectional interactions between oxytocin and dopaminergic systems [159], inhibition of excessive mesolimbic dopamine and modulation of central hypoglutamatergia by oxytocin [37,160] through decreasing intracerebral oxytocin, upregulating oxytocin receptors, and decreasing oxytocin receptor affinity in the amygdala [161] in animal studies consistently supports a therapeutic effect of oxytocin on the positive symptoms of schizophrenia. Increased trust of strangers in healthy subjects after intranasal oxytocin [162] may explain the role of oxytocin in ameliorating paranoid delusions, which involve the mistrust driven by the misattribution of malevolent intentions to others through shifting attributional biases [160]. Direct coupling with the dopaminergic brain network for social reward and reinforcement by oxytocin [163] may attenuate the defensive behavior and alter the social information processing by increasing the salience of social stimuli [20], in line with the studies that demonstrated the modulation of brain reward circuitry by oxytocin administration in humans [26,164]. It is now understood that oxytocin may modulate the inhibitory stress response through an action on the hypothalamic–pituitary–adrenal axis [165] and contribute to the social cognition associated with fear learning. Undoubtedly, the interactions of oxytocin with other functional systems are complex and further work is needed to understand their implications and possible mediations of oxytocin in patients with schizophrenia.

## 12. Conclusions

Schizophrenia is a chronic and debilitating illness accompanied by social function impairment. Dysregulation of oxytocin may play a role in regulating the expression of schizophrenia. It is apparent that the treatment options for schizophrenia need to be improved. Given oxytocin’s role in human behaviors, particularly in social cognition, a variety of studies have examined the potential clinical benefits of oxytocin in improving psychopathology in patients with schizophrenia. Driven by the convergent findings in preclinical studies and the investigation of oxytocin dysregulation in patients with schizophrenia, burgeoning evidence from the double-blind randomized controlled trials demonstrates encouraging results using intranasal oxytocin as an augmentation to antipsychotics in ameliorating both the positive and negative symptoms of schizophrenia. More importantly, intranasal oxytocin may help restore social cognitive deficits in patients with schizophrenia. Across all clinical studies, intranasal oxytocin is well-tolerated and produced almost no adverse effects. In conclusion, oxytocin is a promising candidate for the treatment of schizophrenia in a time of flagging innovation and safety in treatment discovery.

## Figures and Tables

**Table 1:** Studies of oxytocin treatment in patients with schizophrenia stable on antipsychotics.

Study	Study Design	*N*; Population	Group		M:F	Route; Dosing; Duration	Results		
							Positive Symptoms	Negative Symptoms	Cognitive Functions
Abram et al., 2020	dbRCT; crossover	22 OPD; SSD/ 24 HC	SSD		22:0	Intranasal; 40 IU; single dose	N/A	Oxytocin group (only in Sch) increased in rsFC between the amygdala and left MTG/STS/AngG that contributes to negative symptoms *	N/A
			HC		24:0				
Abu-Akel et al., 2014	dbRCT; crossover	28 OPD; Sch / 27 HC	Sch		28:0	Intranasal; 24 IU; single dose	N/A	N/A	Oxytocin group (only in HC) induced an empathy bias toward the conflictual out-group members *, but heightened an empathic bias toward the in-group members when rating non-painful stimuli in Sch
			HC		27:0				
Averbeck et al., 2012	dbRCT; crossover	21 OPD; Sch	Sch		21:0	Intranasal; 24 IU; single dose	N/A	N/A	Oxytocin group improved in hexagon emotion discrimination task *
Bradley et al., 2019	dbRCT; crossover	70 OPD; Sch/ 80 HC	Sch		51:19	Intranasal; 40 IU; single dose	N/A	N/A	Nonsignificant for LNS
			HC		55:25				
Bradley et al., 2019	dbRCT; crossover	33 OPD; Sch/39 HC	Sch		33:0	Intranasal; 40 IU; single dose	N/A	N/A	Oxytocin group increased in fixation time among Sch but decreased among HC *; higher attachment anxiety and greater symptom severity predicted increased fixation time on the eyes
			HC		39:0				
Bradley et al., 2020	dbRCT	37 N/A; Sch/ 51 HC	Sch	Oxytocin	15:0	Intranasal; 40 IU; single dose	N/A	N/A	Both groups increased in bidding, motivated by preferences for both monetary and social reward; less overbidding was in Sch initially but sustained across trials compared with HC *
				Placebo	22:0				
			HC	Oxytocin	25:0				
				Placebo	26:0				
Bradley et al., 2021	dbRCT; crossover	26 OPD; Sch/ 38 HC	Sch		0:26	Intranasal; 40 IU; single dose	N/A	N/A	Nonsignificant for mentalizing skill
			HC		0:38				
Buchanan et al., 2017	dbRCT	56 OPD and 10 IPD; Sch and SchA	Oxytocin		14:2	Intranasal; 24 IU twice daily; 6 weeks	Nonsignificant for BPRS-positive symptoms	Nonsignificant for SANS	Nonsignificant for cognitive impairment measured by MCC scores
			Galantamine		14:6				
			Placebo		17:3				
Cacciotti-Saija et al., 2015	dbRCT	52 OPD; SSD	Oxytocin ^†^		18:9	Intranasal; 24 IU twice daily; 6 weeks	Nonsignificant for SAP	Nonsignificant for SANS; oxytocin group showed that increased use of nasal spray was associated with reductions in negative symptoms	Nonsignificant for Social Functioning Scale, RMET, Facial Expressions of Emotions Task, False Belief Picture Sequencing Task, and Ambiguous Intentions Hostility Questionnaire
			Placebo ^†^		18:7				
Cohen et al., 2017 (see Buchanan et al., 2017)	dbRCT	40 N/A; Sch and SchA	Oxytocin		(10)	Intranasal; 24 IU twice daily; 6 weeks	N/A	Oxytocin groups showed an increase in negative facial expressions across the two role-play tasks but statistically nonsignificant	N/A
			Galantamine		(15)				
			Placebo		(15)				
Dagani et al., 2016	dbRCT; crossover	32 OPD; Sch	Sch		26:6	Intranasal; 40 IU once daily; 4 months	Nonsignificant for PANSS positive subscale	Nonsignificant PANSS negative subscale	Nonsignificant for PANSS general psychopathology
Davis et al., 2013	dbRCT	23 OPD; Sch	Oxytocin		11:0	Intranasal; 40 IU; single dose	N/A	N/A	Oxytocin group improved in high-level social cognition (sarcasm, deception, and empathy) *
			Placebo		12:0				
Davis et al., 2014	dbRCT	27 OPD; Sch	Oxytocin ^¶^		13:0	Intranasal; 40 IU twice weekly; 6 weeks	Nonsignificant for BPRS	Nonsignificant for CAINS	Oxytocin group improved in empathic accuracy *; nonsignificant for MCCB scores
			Placebo ^¶^		14:0				
De Coster et al., 2019	dbRCT; crossover	23 OPD; SSD/ 25 HC	SSD		23:0	Intranasal; 40 IU; single dose	N/A	N/A	Oxytocin group (only in Sch) increased in accuracy and right temporo-parietal junction activation for false belief task (belief) and person description task (thought and emotion) *
			HC		25:0				
Dwyer et al., 2020	dbRCT	40 OPD; Sch and SchA	Oxytocin		10:2	Intranasal; 24 IU twice daily; 6 weeks	N/A	N/A	Nonsignificant for social skills, linguistic content, and self-reported affiliation and affect during a roleplay interaction
			Galantamine		11:3				
			Placebo		12:2				
Feifel et al., 2010	dbRCT; crossover	15 OPD; Sch	Sch		12:3	Intranasal; 40 IU twice daily; 3 weeks	Oxytocin group significantly improved in PANSS positive subscale *	Oxytocin group significantly improved in PANSS negative subscale *	Nonsignificant for PANSS general psychopathology
Feifel et al., 2012 (see Feifel et al., 2010)	dbRCT; crossover	15 OPD; Sch	Sch		12:3	Intranasal; 40 IU twice daily; 3 weeks	N/A	N/A	Oxytocin group improved in California Verbal Learning Test but not LNS *
Fischer-Shofty et al., 2013a	dbRCT; crossover	35 N/A; Sch/ 48 HC	Sch		31:4	Intranasal; 24 IU; single dose	N/A	N/A	Oxytocin group (both Sch and HC) improved in Interpersonal Perception Task but only Sch group improved in recognition of kinship *
			HC		39:9				
Fischer-Shofty et al., 2013b	dbRCT; crossover	30 N/A; Sch/ 35 HC	Sch		27:3	Intranasal; 24 IU; single dose	N/A	N/A	Both groups were more accurate in recognizing fearful facial expressions
			HC		32:3				
Fulford et al., 2018	dbRCT; crossover	42 OPD; Sch / 43 HC	Sch		29:13	Intranasal; 40 IU; single dose	N/A	N/A	Nonsignificant for social vigor task
			HC		31:12				
Gibson et al., 2014	dbRCT	14 OPD; Sch	Oxytocin		6:2	Intranasal; 24 IU twice daily; 6 weeks	Both groups significantly improved in PANSS positive subscale	Oxytocin group improved in PANSS negative subscale	Both groups significantly improved in PANSS general psychopathology and Brüne total score; only oxytocin group improved in fear recognition and perspective-taking
			Placebo		5:1				
Goldman et al., 2011	dbRCT, crossover (3 arms)	13 OPD; Sch/ 11 HC	Sch (Pd)		3:2	Intranasal; 10 IU or 20 IU; single dose	N/A	N/A	(Only in Sch) 10 IU dose decreased emotional recognition due to an increased propensity to identify all emotions regardless of whether they were displayed *; 20 IU dose improved emotional recognition around fear recognition in Pd than NPd
			Sch (NPd)		4:4				
			HC		4:7				
Guastella et al., 2015	dbRCT; crossover	21 OPD; Sch and SchA	Sch/SchA		21:0	Intranasal; 40 IU; single dose	N/A	N/A	Oxytocin group improved in performance on higher-order social cognition tasks (the hinting task and the non-faux condition of FPRT) but no effects on general neurocognition *
Halverson et al., 2019 (see Jarskog et al., 2017)	dbRCT	62 OPD; Sch and SchA	Oxytocin		24:8	Intranasal; 24 IU twice daily; 12 weeks	N/A	N/A	Oxytocin group improved interpersonal reactivity index perspective-taking; nonsignificant for self-reported symptoms, empathy, or introspective accuracy
			Placebo		23:7				
Horta de Macedo et al., 2014	dbRCT; crossover	20 OPD; Sch/ 20 HC	Sch		20:0	Intranasal; 48 IU; single dose	Nonsignificant for BPRS	Nonsignificant for PANSS negative subscale	Nonsignificant for facial emotion matching task
			HC		20:0				
Jarskog et al., 2017	dbRCT	62 OPD; Sch and SchA	Oxytocin		24:8	Intranasal; 24 IU twice daily; 12 weeks	Both groups significantly improved in PANSS positive subscale	Oxytocin group improved in PANSS negative subscale; only schizophrenia group showed significant between-group improvement *	Both groups significantly improved in Brüne total scores
			Placebo		23:7				
Lee et al., 2013	dbRCT	16 OPD and 12 IPD; Sch	Oxytocin		9:4	Intranasal; 20 IU twice daily; 3 weeks	Oxytocin group not improved compared with placebo	Oxytocin inpatient subgroup showed improvement	Oxytocin group significantly improved in olfactory identification *
			Placebo		11:4				
Lee et al., 2019	dbRCT	28 OPD or IPD; Sch and SchA	Oxytocin		9:4	Intranasal; 20 IU twice daily; 3 weeks	N/A	N/A	Nonsignificant for Mayer-Salovay Caruso Emotional Intelligence Test and Maryland Assessment of Social Competence
			Placebo		11:4				
Marotta et al., 2020	RCR	5 IPD; TRS with clozapine	Oxytocin		N/A	Sublingual; 10 IU once daily to 20 IU thrice daily; at least 1 year	Augmented with oxytocin maintained lowered positive symptoms	Augmented oxytocin reduced negative symptoms	Augmented oxytocin increased occupational and social functioning
Michalopoulou et al., 2015	dbRCT; crossover	21 N/A; Sch	Sch		21:0	Intranasal; 24 IU; single dose	N/A	N/A	Oxytocin group improved in digits backward but not digit symbol coding *
Modabbernia et al., 2013	dbRCT	40 IPD; Sch with risperidone	Oxytocin		17:3	Intranasal; 40 IU twice daily; 8 weeks	Oxytocin group significantly improved in PANSS positive subscale *	Oxytocin group significantly improved in PANSS negative subscale *	Oxytocin group significantly improved in PANSS general psychopathology subscale *
			Placebo		16:4				
Ota et al., 2018	Open-label	16 N/A; Sch	Oxytocin		7:9	Intranasal; 12 IU twice daily; 3 months	Oxytocin significantly improved in PANSS positive subscale *	Oxytocin significantly improved in PANSS negative subscale (blunted affect, emotional withdrawal, lack of spontaneity, and flow of conversation) *	Oxytocin significantly improved in PANSS general psychopathology and increased in category fluency of BACS *
Pedersen et al., 2011	dbRCT	10 OPD and 10 IPD; Sch	Oxytocin		9:2	Intranasal; 24 IU twice daily; 2 weeks	Oxytocin group significantly improved in PANSS positive subscale *	Nonsignificant PANSS negative subscale	Oxytocin group significantly improved in PANSS general psychopathology subscale and accurate identification of second-order false belief in the Brüne Task *
			Placebo		8:1				
Porffy et al., 2020	dbRCT; crossover	19 OPD; Sch and SchA	Sch		19:0	Intranasal; 40 IU; single dose	N/A	N/A	Oxytocin group increased in the total number of fixations, dispersion, and saccade amplitudes, while decreasing the duration of fixations *
Shin et al., 2015	dbRCT; crossover	16 OPD; Sch / 16 HC	Sch		16:0	Intranasal; 40 IU; single dose	N/A	N/A	Oxytocin group (Only in Sch) decreased in amygdala activity for fearful emotion and increased in activity for happy faces *
			HC		16:0				
Singh et al., 2016	dbRCT; crossover	17 N/A; Sch / 15 HC	Sch		9:8	Intranasal; 24 or 48 IU; single dose	N/A	N/A	Oxytocin group enhanced mu-suppression at doses of 48 IU in Sch male *
			HC		6:9				
Strauss et al., 2019	dbRCT	62 OPD; Sch	Oxytocin ^‡^		18:13	Intranasal; 36 IU twice daily; 24 weeks	N/A	N/A	Nonsignificant for RMET, empathic accuracy task, trust game, and facial emotion recognition test
			Placebo ^‡^		20:11				
Warren et al., 2018	dbRCT; crossover	16 N/A; Sch	Sch		8:8	Intranasal; 24 IU; single dose	N/A	N/A	Oxytocin group decreased in leptin but nonsignificant for self-reported satiety or test meal consumption, insulin or glucose levels, or sensory measures *
Woolley et al., 2014	dbRCT; crossover	29 N/A; Sch and SchA/ 31 HC	Sch / SchA		29:0	Intranasal; 40 IU; single dose	N/A	N/A	Oxytocin group (only in Sch) improved in controlled social cognition *
			HC		31:0				
Woolley et al., 2015	dbRCT; crossover	31 OPD; SSD/ 34 HC	Sch		25:6	Intranasal; 40 IU; single dose	N/A	N/A	Oxytocin group improved detection of lyral but not anise in Sch than in HC *
			HC		32:2				
Woolley et al., 2017	dbRCT; crossover	33 N/A; SSD / 35 HC	SSD		30:3	Intranasal; 40 IU; single dose	N/A	Oxytocin group (only in Sch) increased in facial expressivity *	N/A
			HC		33:2				
Wynn et al., 2019	dbRCT; crossover	47 OPD; Sch	Sch		31:16	Intranasal; 8, 12, 24, 36, 48, 60, 72, or 84 IU; single dose	N/A	N/A	Oxytocin group enhanced mu-suppression at doses of 36 and 48 IU*

* Test for between-group change is statistically significant. ^‡^ Participants received oxytocin or placebo 45 min prior to each twice-weekly session on cognitive-behavioral social skills training. ^†^ Participants received oxytocin or placebo 15 min prior to each weekly session on social cognitive training. ^¶^ Participants received oxytocin or placebo 30 min prior to each weekly session on social cognitive skills training.

## Abbreviations

dbRCT, Double-Blind Randomized Controlled Trial; RCR, Retrospective Chart Review; Sch, Schizophrenia; SchA, Schizoaffective Disorder; SSD, Schizophrenia Spectrum Disorder; Pd, Polydipsia; NPd, Nonpolydipsia; HC, Healthy Controls; N/A, Not Applicable; OPD, Outpatients; IPD, Inpatients; M, Male; F, Female; IU, International Unit; PANSS, Positive and Negative Syndrome Scale; SAPS, Scale for the Assessment of Positive Symptoms; SANS, Scale for the Assessment of Negative Symptoms; BPRS, Brief Psychiatric Rating Scale; RMET, Reading the Mind in the Eyes Test; FPRT, Faux Pas Recognition Task; LNS, Letter Number Sequence; CAINS, Clinical-Assessment Interview for Negative Symptoms; BACS, Brief Assessment of Cognition in Schizophrenia; MCCB MATRICS Consensus Cognitive Battery composite; MTG/STS/AngG, Middle Temporal Gyrus, Superior Temporal Gyrus, and Angular Gyrus, respectively; rsFC, Resting-State Functional Connectivity.
